# Expanding the Toolkit for Structural Cell Biology with ExoSloNano

**DOI:** 10.1063/4.0000823

**Published:** 2025-10-27

**Authors:** Lindsey N Young, Alice Sherrard, Huabin Zhou, Farhaz Shaikh, Joshua Hutchings, Margo Riggi, Mythreyi Narasimhan, Eric Bennett, Michael Rosen, Antonio Giraldez, Elizabeth Villa

**Affiliations:** 1 UCSD, La Jolla, CA, USA; 2 Yale University, New Haven, CT, USA; 3 UT Southwestern, Dallas, TX, USA; 4 UCSF, San Francisco, CA, USA; 5 Chan Zuckerberg, Redwood City, CA, USA; 6 MPI, Munich, Bavaria, Germany; 7 HHMI, Dallas, TX, USA; 8 HHMI, La Jolla, CA, USA

## Abstract

In situ cryo-Electron Microscopy (cryo-EM) enables the direct interrogation of structure-function relationships by resolving macromolecular structures in their native cellular environment. Tremendous progress in sample preparation, imaging and data processing over the past decade has contributed to the identification and determination of large biomolecular complexes. However, the majority of proteins are of a size that still eludes identification in cellular cryo-EM data, and most proteins exist in low copy numbers. Therefore, novel tools are needed for cryo-EM to identify the vast majority of macromolecules across multiple size scales (from microns to nanometers). Here, we introduce and validate novel nanogold probes that enable the detection of specific proteins using cryo-ET (cryo-Electron Tomography) and resin-embedded correlated light and electron microscopy (CLEM). We demonstrate that these nanogold probes can be introduced into live cells, in a manner that preserves intact molecular networks and cell viability. We use this system to identify both cytoplasmic and nuclear proteins by room temperature EM, and resolve associated structures by cryo-ET. We further employ gold particles of different sizes to enable future multiplexed labeling and structural analysis. By providing high efficiency protein labeling in live cells and molecular specificity within cryo-ET tomograms, we establish a broadly enabling tool that significantly expands the proteome available to electron microscopy.

